# Optimization and Validation of RP-HPLC-UV/Vis Method for Determination Phenolic Compounds in Several Personal Care Products

**DOI:** 10.1155/2011/858153

**Published:** 2011-06-23

**Authors:** Mohammed Akkbik, Zaini Bin Assim, Fasihuddin Badruddin Ahmad

**Affiliations:** Department of Chemistry, Faculty of Resource Science and Technology, University Malaysia Sarawak, 94300 Kota Samarahan, Sarawak, Malaysia

## Abstract

An HPLC method with ultraviolet-visible spectrophotometry detection has been optimized and validated for the simultaneous determination of phenolic compounds, such as butylated hydroxyanisole (BHA) and butylated hydroxytoluene (BHT) as antioxidants, and octyl methyl cinnamate (OMC) as UVB-filter in several personal care products. The dynamic range was between 1 to 250 mg/L with relative standard deviation less than 0.25% (*n* = 4). Limits of detection for BHA, BHT, and OMC were 0.196, 0.170, and 0.478 mg/L, respectively. While limits of quantification for BHA, BHT, and OMC were 0.593, 0.515, and 1.448 mg/L, respectively. The recovery for BHA, BHT, and OMC was ranged from 92.1–105.9%, 83.2–108.9%, and 87.3–103.7%, respectively. The concentration ranges of BHA, BHT, and OMC in 12 commercial personal care samples were 0.13–4.85, 0.16–2.30, and 0.12–65.5 mg/g, respectively. The concentrations of phenolic compounds in these personal care samples were below than maximum allowable concentration in personal care formulation, that is, 0.0004–10 mg/g, 0.002–5 mg/g, and up to 100 mg/g for BHA, BHT, and OMC, respectively.

## 1. Introduction

**Figure 1:**
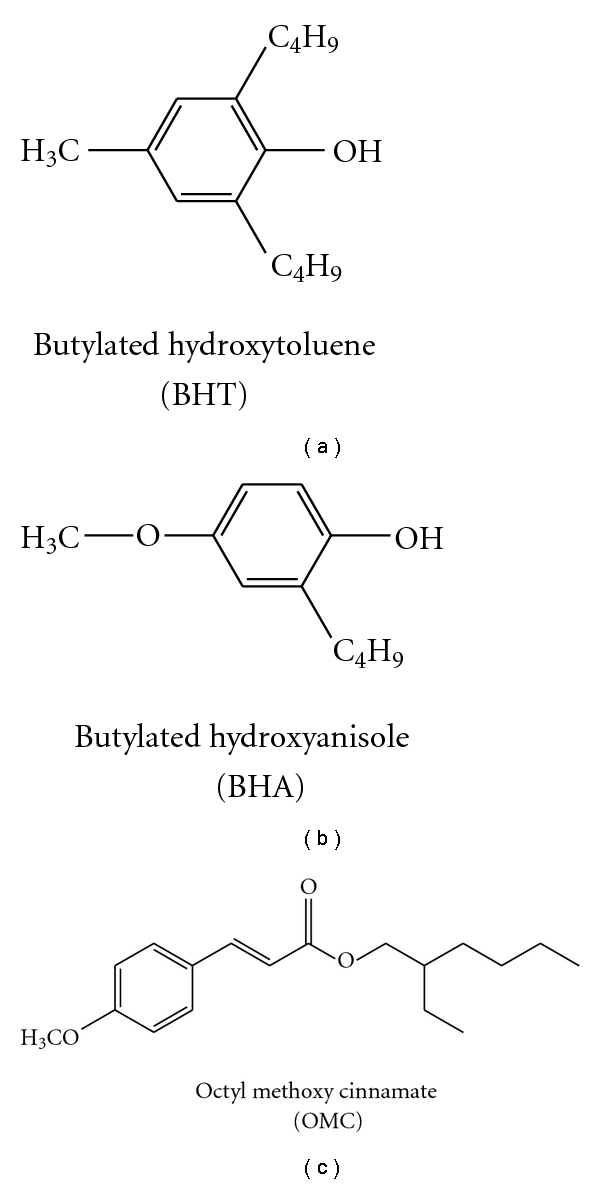
Structures of common phenolic compounds in personal care products.

Phenolic compounds such as butylated hydroxyanisole (BHA) and butylated hydroxytoluene (BHT) act as antioxidants and octyl methyl cinnamate (OMC) as UVB-filter are active compounds in personal care products (see Figure 1) [1, 2].

BHA and BHT are addedsingly or in combination to prevent oxidative rancidity in personal care products [3]. While octyl methyl cinnamate (OMC) is used to absorb the dangerous UV-light between 280–320 mmto and to protect the skin from sunburn [2]. The concentration of BHA and BHT in personal care formulation depends on the amount of sensitive compounds (alpha hydroxy acids, ceramides, lipids, vitamins, oils, and so forth) that are susceptible to oxidation by the oxygen in the atmosphere making it possible for the unstable peroxide radicals [4, 5]. BHA and BHT are able to inhibit reactions promoted by oxygen, thus avoiding the oxidation and are intended to prevent the appearance of ketones and aldehydes that can give a product a disagreeable smell and rancidity [5]. To prevent cosmetic formulations from peroxide radicals we must use antioxidant compounds which have the ability to neutralize those radicals through the transfer of hydrogen to this radical, stabilizing the antioxidant by resonance [6, 7]. While the concentration of OMC depends on the type of product and part of body it is applied on (face, hand, lips, and other parts of human body) [2, 8–11]. 

Reversed phase HPLC with UV/Vis detector (RP-HPLC-UV/Vis) is an important analytical technique with strong chromophores that absorb light in the wavelength region from 200 nm to 800 nm [12]. Numerous publications and research papers focus on separation methods to detect phenolic antioxidants as BHA and BHT and phenolic UVB-filte as OMC in personal care products using RP-HPLC-UV/Vis [2, 5, 13]. The objective of this study is to determine the optimum analysis condition and validate the method for a simultaneous detection, identification, and quantification of phenolic compounds as well as to develop an analytical method for evaluation and quality control of phenolic compounds by RP-HPLC-UV/Vis in personal care products. 

## 2. Experimental

### 2.1. Personal Care Samples

12 personal care samples were collected from local supermarket in Kuching city. Four types of personal care products were collected, that is, sunscreen cream, milk lotion, hair gel, and hair oil. The personal care samples were manufactured in Malaysia, Thailand, Indonesia, and Philippines.

### 2.2. Chemicals

All chemical reagents used for analysis phenolic compounds by RP-HPLC-UV/Vis were analytical Grade (99.99%) of Merck (Darmstadt, Germany). The reagents include n-hexane, methanol, ethanol, and acetonitrile. Reverse-osmosis type quality water was used during analysis. Standards of butylated hydroxyanisole BHA (96%), butylated hydroxytoluene BHT (99.8%), and OMC octyl methoxy cinnamate (98%) were purchased from Acros-Organics (New Jersey, USA).

### 2.3. Preparation of Standard Solution

An individual of 5000 mg/L stock solution of BHA, BHT, and OMC in acetonitrile was prepared by weighing equivalent accurately 1250 mg each of BHA, BHT, and OMC in the flask and diluted with 100 mL acetonitrile. The mixture was shaken until a homogenous and clear solution formed and added with acetonitrile until final volume of 250 mL. The stock solution was covered with aluminum foil and stored in a freezer (4°C) and away from light for a maximum of one month. Prior to analysis, standard working solutions were prepared by diluting appropriate amounts of the stock solutions in acetonitrile.

### 2.4. Extraction Procedure

Extraction of BHA, BHT, and OMC from cosmetic samples was performed according to method described by Capitán-Vallvey et al. [4, 5] with slight modification. Briefly, 0.1 to 1 g personal care samples were accurately weighed in the 100 mL capacity round bottom flask. Prior to extraction, 25 mL n-hexane was added to the samples in order to remove lipids, fatty acids, and volatile oils and followed by addition 25 mL acetonitrile. The sample was then extracted by refluxing for 30 minutes at 70°C and stirring. Extraction was performed in triplicates. The crude extract was transferred to separatory funnel, and two layers were formed, that is, n-hexane and acetonitrile phases. The n-hexane phase was repartitioned for two or three times using 10 mL of acetonitrile and shaken vigorously. The n-hexane phase was removed, and acetonitrile phase was collected. The extract (acetonitrile phase) was concentrated using a vacuum rotary evaporator at 45°C. The residue was redissolved with 10 mL of acetonitrile and filtered by membrane filters (Millipore, 0.5 *μ*m × 45 mm) then transferred into a 25 mL volumetric. It was diluted to 25 mL with acetonitrile.

### 2.5. HPLC Analysis

The quantitative and qualitative analysis of phenolic compounds was performed on Shimadzu HPLC system model LC-20AT equipped with four pumps and Shimadzu SPD-20 AV UV/Vis detector. 50 *μ*L samples was injected, and the chromatographic separation was performed on aRP-C_18_ Metacil (5 *μ*m) ODS column, 4.6 mm × 250 mm. The HPLC analysis condition based on the report of Saad et al. [14] with slight modification using 280 nm as maximum wave length (*λ*_max_), acetonitrile (phase A), and (water/acetic acid, 99 : 1, v/v) (phase B) as mobile phase and 0.8 mL/min as flow rate.

## 3. Results and Discussion

### 3.1. Optimization of HPLC Condition

#### 3.1.1. Determination the Optimum Wave Length by Spectrophotometer UV/Vis

The UV spectrum of BHA, BHT, and OMC exhibited maximum absorption at 290, 275 and 300 nm, respectively. For the RP-HPLC analysis, the UV/Vis detector was fixed at 280 nm as maximum wave length (*λ*_max_) for simultaneous determination.

#### 3.1.2. Effect of the pH of Mobile Phase on Resolution Factor (*R*_*s*_)

**Figure 2:**
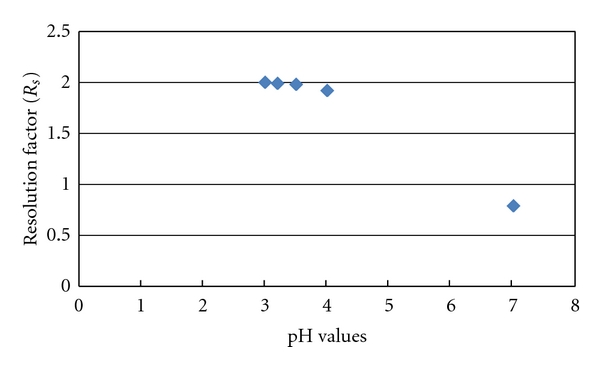
Variation of resolution factor between BHT and OMC at different pH values of phase B of mobile phase.

pH is an important parameter to be optimized as it affects the ionization of phenolic compounds. Separation of BHA, BHT, and OMC are sensitive to the pH values because at low pH values, phenolic antioxidants are ionized due to the increase of protonation in mobile phase [14–17]. The analytical conditions were used for analysis BHA, BHT, and OMC based on the recent report by Saad et al. [14], mixture phase A (acetonitrile) with phase B (water:acetic acid) as mobile phase, 280 nm as maximum wave length, and 0.8 mL/min as flow rate of mobile phase. The pH was optimized by varying the percentage of acetic acid in order to adjust pH of the phase B of mobile phase at pH 3, 3.2, 3.5, 4 and 7, respectively. Decreasing pH value increases the separation and ionization of BHA, BHT, and OMC, especially between BHT, and OMC.  Figure 2 shows the effect of pH on the resolution factor (*R*_*s*_, between BHT, and OMC) by varying the percentage of acetic acid in phase B of mobile phase from 0% to 2% (see Table 1). 

**Figure 3:**
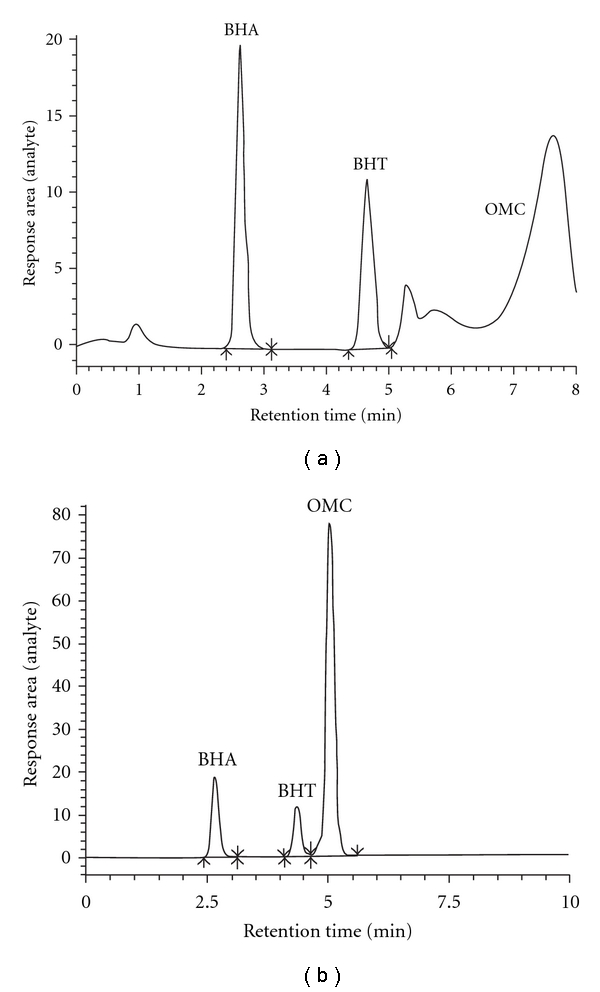
Chromatogram of BHA, BHT, and OMC analyzed using RP-HPLC-UV/Vis at *λ*_max_ = 280 nm (A: pH 7, *R*_*s*_: 0.79 < 1.5 and B: pH 3.5, *R*_*s*_: 1.98 > 1.5).

It was observed that the resolution factor (*R*_*s*_) particularly for separation between BHT and OMC depends on the pH values of phase B of mobile phase. Mixture of water:acetic acid (99 : 1; v/v) of phase B as buffer solution at pH 3.5 was chosed after a compromise between resolution factors (*R*_s_: 1.98 > 1.5) and total time of elute of BHA, BHT, and OMC (5.5 minutes). BHA, BHT, and OMC at pH 3.5 elute earlier compared to at pH 4 and 7 (see Figure 3). The resolution factor was also better at pH 3.5 (*R*_*s*_: 1.98 > 1.5) compared to pH 4 (*R*_*s*_: 1.92 > 1.5) and pH 7 (*R*_*s*_: 0.79 < 1.5).

#### 3.1.3. Effect the Flow Rate of Mobile Phase on Retention Time

Flow rate of mobile phase has important effect on retention time, and peak area and little effect on separation for BHA, BHT, and OMC.  Table 2 shows gradient scaling of flow rates from 0.1 mL/min to 1.25 mL/min using RP-HPLC-UV/Vis at 280 nm with mixture of phase A (acetonitrile) and phase B (water:acetic acid; 99 : 1; v/v) as mobile phase.

#### 3.1.4. Effect of Mobile Phase Composition on Retention Time

**Figure 4:**
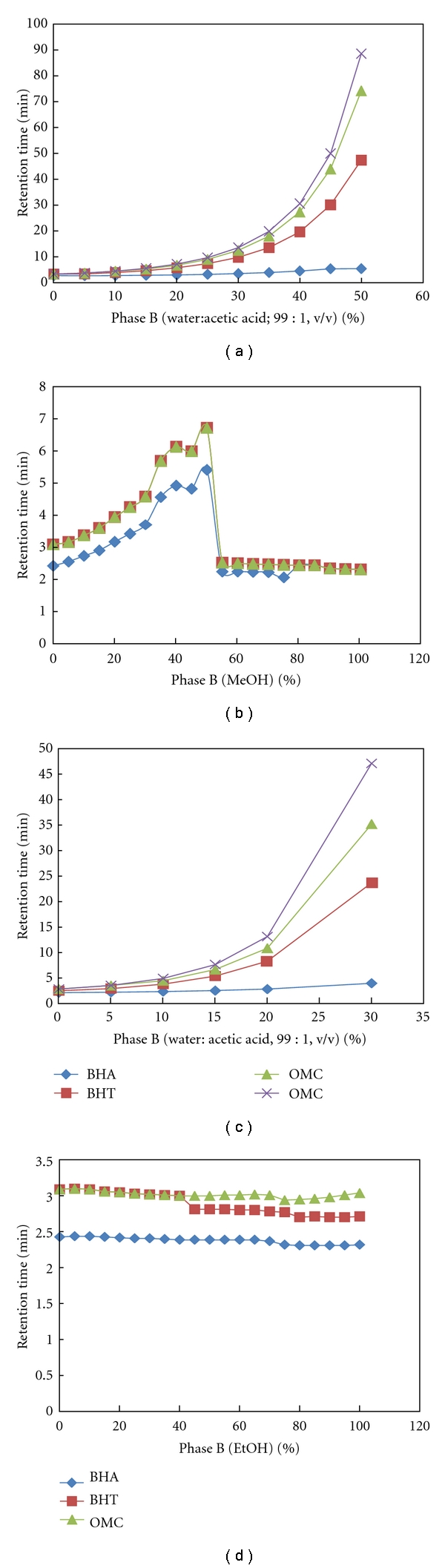
Effect of mobile phase composition on retention time of BHA, BHT, and OMC.

Figure 4 shows that the optimum composition of mobile phase was determined by comparing the influence of different binary mixtures were used in previous studies on retention times of BHA, BHT, and OMC using RP-HPLC-UV/Vis such as acetonitrile with mixture of water:acetic acid (99 : 1; v/v) (a) [14, 18], acetonitrile with methanol (b) [15, 19], ethanol with mixture of water:acetic acid (99 : 1; v/v) (c) [4, 11] and acetonitrile with ethanol (d) [20] at 280 nm as maximum wave length (*λ*_max_) and 0.8 mL/min as flow rate of mobile phase.

## 4. Validation Method

The validation study for BHA, BHT, and OMC using RP-HPLC-UV/Vis was performed under the optimized conditions at 280 nm as maximum wave length, 0.8 mL/min as flow rate of mobile phase, and mixture phase A (acetonitrile) with phase B (water:acetic acid; 99 : 1; v/v) as mobile phase with elution ratio (90A : 10B; v/v) during the analysis time (8 minutes).

### 4.1. Linearity and Limits of Detection (LOD) and Quantification (LOQ)

**Figure 5:**
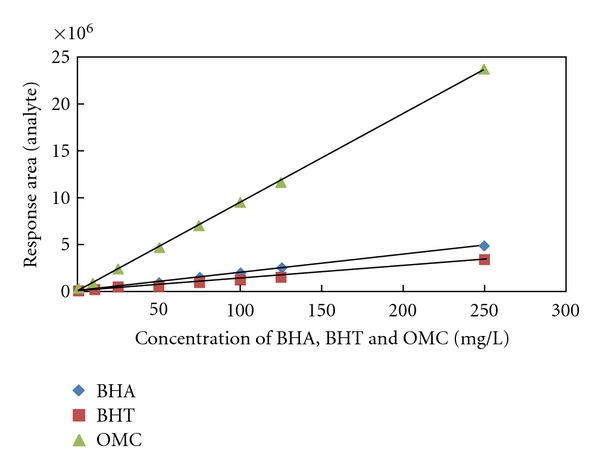
Calibration curves for BH, BHT, and OMC analysed on RP-HPLC-UV/Vis at *λ*_max_ = 280 nm, 0.8 mL/min and (water: acetic acid, 99 : 1, v/v) as mobile phase.

Eight standards solution of BHA, BHT, and OMC in acetonitrile concentrations of 1, 10, 25, 50, 75, 100, 125, and 250 mg/L were prepared. The calibration curves obtained by plotting the peak area of chromatograms for BHA, BHT, and OMC against the concentration are presented in Figure 5, with four replicates (*n* = 4). Correlation coefficients (*R* ^2^) were 0.999 for all standards.  Table 3 shows the validation of analytical method obtained from the calibration curves of BHA, BHT, and OMC analysed on RP-HPLC-UV/Vis.

LOD for BHA and BHT by RP-HPLC-UV/Vis in this study (0.196 and 0.170 mg/L, resp.) are low compared with previous publications for LOD of BHA and BHT reported by Capitán-Vallvey et al. [5] (1.8 and 2.1 mg/L, resp.), and by Saad et al. [14] (0.5 and 0.5 mg/L, resp.), by Campos and Toledo [21] (0.6 and 2.7 mg/L, resp.), by Perrin and Meyer [22] (2 and 2 mg/L, resp.). While, LOD for OMC by RP-HPLC-UV/Vis in this study (0.478 mg/L) is low compared with previous publications for LOD value of OMC have reported by Chawla and Mrig [2] (1.38 mg/L), Salvador and Chisvert [11] (0.9 mg/L), De Orsi et al. [15] (0.8 mg/L) and Mazonakis et al. [23] (1.11 mg/L). Thus, the LOD for BHA, BHT, and OMC in this study are better compared to previous studies.

### 4.2. Recovery Efficiency and Method Performance

The relative recoveries for phenolic compounds were determined by using the external standard additions methodology at four spiked levels 1, 5, 10, and 25 mg/L by comparison with a standard chromatogram of similar concentration. Mean recoveries for every spiked level were determined at three times with four replicates representing at each time (see Table 4).

The recovery ranges of BHA and BHT in this study (92.1%–105.9%, 83.2%–108.9%, resp.) are better than previous paper by Saad et al. [14] (96.7%–101.2%, 73.9%–94.6%, resp.) using the external standard addition methodology. While, the recovery range of OMC in this study (87.3%–103.7%) is similar with earlier study reported by Mazonakis et al. [23] (87.6%–101.3%).

### 4.3. Analysis Real Samples

Four types of personal care products such as sunscreen cream, milk lotion, hair gel and hair oil with three different samples for every type were analyzed for their BHA, BHT, and OMC content as can be seen in Table 5. Every real sampleswere analysed three times with four replicates for each time. 

**Figure 6:**
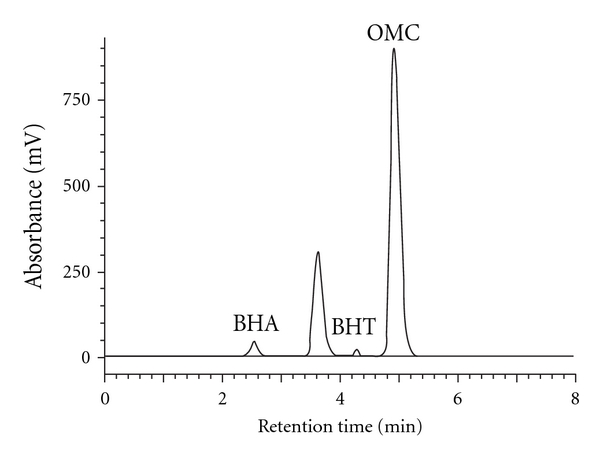
Chromatogram of BHA, BHT, and OMC in Aiken sunscreen cream sample using RP-HPLC-UV/Vis at *λ*_max_ = 280 nm.

**Figure 7:**
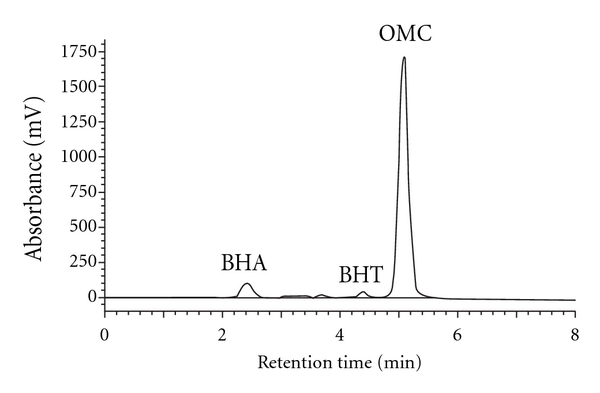
Chromatogram of BHA, BHT, and OMC in Nivea milk lotion sample using RP-HPLC-UV/Vis at *λ*_max_ = 280 nm.

Table 5 shows that concentration ranges of BHA and BHT in three different commercial products of sunscreen cream, namely Aiken, Nivea and Gervenne (1.82–4.85 and 1.01–1.33 mg/g, resp.) are higher than concentration range of BHA and BHT in other commercial sunscreen products reported by Yang et al. [3] (0.003–0.026 and 0.006 mg/g, resp.) (Figures 6 and 7). While the concentration of BHT in these sunscreen products (1.01–1.33 mg/g) is lower than the concentration of BHT in other commercial products of sunscreen products reported by Capitán-Vallvey et al. [4] (2.263 mg/g). On the other hand, the concentration range of OMC in these sunscreen products (16.23–65.50 mg/g) is low compared with previous studies for concentration range of OMC in other commercial sunscreen products reported by Chawla and Mrig [2] (56.12–91.02 mg/g), Wang and Chen [8] (18.3–80.1 mg/g), Chisvert et al. [9] (19.5–90.5 mg/g), De Orsi et al. [15](20–74 mg/g), and Chisvert et al. [24] (5.8–77.8 mg/g).

Table 5 shows that concentration ranges of BHA and BHT in three different commercial products of milk lotion, namely, Nivea, New Trendy, and Garnier (2.74–450 and 0.73–2.30 mg/g, resp.) are high comparedwith previous studies for concentration range of BHA and BHT in other commercial products of milk lotion reportedby Yang et al. [3] (not detected and not detected), Capitán-Vallvey et al. [4] (0.127 and 0.610 mg/g), Capitán-Vallvey et al. [5] (not detected and 0.408 mg/g) and Tsai and Lee [25] (not detected and not detected). The concentration range of OMC in these milk lotion samples (8.99–17.00 mg/g) are low compared with previous studies for concentration range of OMC in other commercial products of milk lotion reported by Salvador and Chisvert [11] (30.2–74.1 mg/g) and Mazonakis et al. [23] (70–75 mg/g).

**Figure 8:**
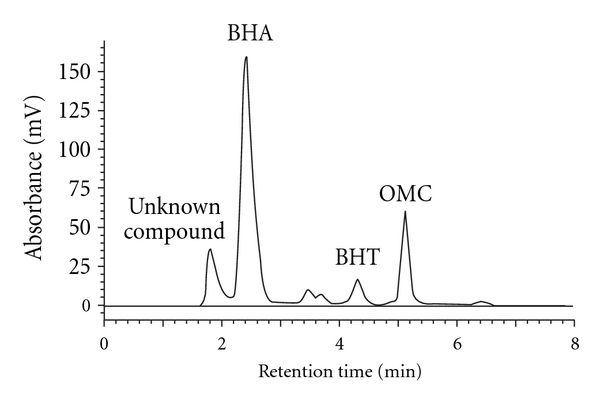
Chromatogram of BHA, BHT, and OMC in De Boy hair gel sample using RP-HPLC-UV/Vis at *λ*_max_ = 280 nm.

**Figure 9:**
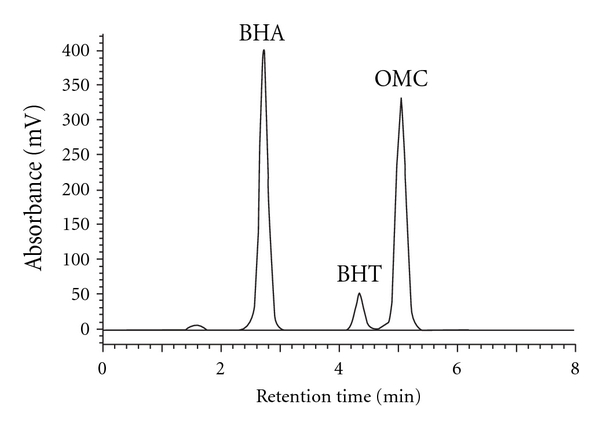
Chromatogram of BHA, BHT, and OMC in Elite hair oil sample using RP-HPLC-UV/Vis at *λ*_max_ = 280 nm.

Table 5 shows concentration ranges of BHA, BHT, and OMC in three different hair gel products, namely, De Boy, Beyond, and Elite (1.28–1.51 and 0.16–0.22 mg/g, resp.) are high compare with previous studies for concentration range of BHA and BHT in other commercial hair gel samples reported by Yang et al. [3] (not detected and not detected, resp.) and García-Jiménez et al. [26] (not detected and not detected, resp.) (Figures 8 and 9). While the concentration range of OMC in these hair gel samples (0.12–0.84 mg/g) is higher than the concentration of OMC in other commercial hair care products reported by Gao and Bedell [27] (not detected).

Table 5 shows concentration ranges of BHA and BHT in three different commercial hair oil products, namely, Elite, Gervenne and Johnsons (0.13–3.89 and 0.18–1.54 mg/g, resp.) is high compared with previous studies for concentration of BHA and BHT in other commercial products of hair oils reported by Capitán-Vallvey et al. [4] (0.031 and 0.100 mg/g, resp.) and Capitán-Vallvey et al. [5] (not detected and 0.659 mg/g, resp.). While the concentration range of OMC in these hair oil samples (0.57–3.40 mg/g) is higher than the concentration of OMC in other commercial products of hair oil reported by Fent et al. [28] (not detected).

## 5. Conclusion

The analytical method by RP-HPLC-UV/Vis in this study is modern for simultaneous determination of common phenolic compounds in personal care products. The optimum parameters that can be used are as follows; binary mixture of phase A (acetonitrile) and phase B (water/acetic acid, 99 : 1, v/v) as mobile phase with elution ratio (90 A: 10 B, v/v) during the analysis time (8 minutes), pH 3.5 of phase B (using acetic acid for adjust it), 0.8 mL/min as flow rate and 280 nm as maximum wave length. The satisfactory results of optimization and validation methods are quick, accurate, sensitive, excellent recoveries, convenient and effective for phenolic compounds. The developed method was successfully applied to fingerprint analysis of personal care products as well as quantify the relevant phenolic compounds markers present in these products under optimum parameters. This method can be applied to analyze the phenolic compounds in commercial cosmetic and food products.

## Figures and Tables

**Table 1:** Effect of acetic acid percentage in phase B of mobile phase on pH, resolution factors, and total analysis time.

Acetic acid concentration (%, v/v)	0	0.5	1	1.5	2
pH value	7	4	3.5	3.2	3
Resolution factors (*R*_*s*_)	0.79	1.92	1.98	1.99	2
Total time of elute the analytes (minutes)	8.5	6.0	5.5	5.3	5.3

**Table 2:** The retention times of BHA, BHT, and OMC at different flow rate of mobile phase.

Flow rate (mL/min)	Retention time of BHA (minutes)	Retention time of BHT (minutes)	Retention time of OMC (minutes)
0.10	21.18	34.93	40.69
0.15	13.98	22.81	26.48
0.20	10.53	16.89	19.49
0.25	8.59	14.49	16.99
0.30	7.02	11.22	12.94
0.35	5.90	9.09	10.44
0.40	5.34	8.86	9.93
0.45	4.97	8.08	8.92
0.50	4.3	6.74	7.74
0.55	3.82	6.05	6.95
0.60	3.49	5.51	6.33
0.65	3.21	5.03	5.79
0.70	3.03	5.03	5.85
0.75	2.82	4.60	5.33
0.80	2.65	4.35	5.05
0.85	2.35	3.79	4.37
0.90	2.33	3.72	4.29
0.95	2.22	3.63	4.19
1.00	2.09	3.29	3.79
1.05	1.97	3.06	3.62
1.10	1.92	3.05	3.58
1.15	1.87	3.01	3.56
1.20	1.81	2.94	3.48
1.25	1.72	2.85	3.29

**Table 3:** Validation of analytical method for BHA, BHT, and OMC by RP-HPLC-UV/Vis.

Compound	Retention time (minutes)	Calibration equation	*R* ^2^	RSD%	LOD (mg/L)	LOQ (mg/L)
BHA	2.60	*y* =19673*x* + 2579	0.999	0.18	0.196	0.593
BHT	4.35	*y* = 13410*x* − 5551	0.999	0.17	0.170	0.515
OMC	4.95	*y* = 95019*x* − 14004	0.999	0.25	0.478	1.448

**Table 4:** Results of recovery study for BHA, BHT, and OMC by RP-HPLC-UV/Vis at *λ*_max_ = 280 nm.

Relative recovery (%, *n* = 12)						
Spiked (mg/L)	BHA	RSD%	BHT	RSD%	OMC	RSD%
1	105.9	2.64	108.9	7.69	103.7	2.53
5	102.3	3.72	102.8	4.02	94.6	1.95
10	99.7	1.65	95.9	3.13	93.3	1.45
25	92.1	1.18	83.2	2.24	87.3	1.27

**Table 5:** Concentration of BHA, BHT, and OMC in sunscreen cream, milk lotion, hair gel, and hair oil samples determined by RP-HPLC-UV/Vis at *λ*_max_ = 280 nm.

Type	Commercial name	Country of origin	Phenolic compounds	Mean concentration (mg/g)				
				(1) (*n* = 4)	(2) (*n* = 4)	(3) (*n* = 4)	Average (mg/g)	RSD%
Sunscreen cream	Aiken	Malaysia	BHA	4.80 ± 0.10	4.90 ± 0.07	4.90 ± 0.05	4.85	1.50
			BHT	1.30 ± 0.06	1.40 ± 0.07	1.28 ± 0.03	1.33	3.88
			OMC	62.1 ± 0.60	65.9 ± 0.41	68.5 ± 0.51	65.5	0.77
	Nivea	Thailand	BHA	3.31 ± 0.09	3.03 ± 0.08	3.43 ± 0.07	3.26	2.43
			BHT	1.16 ± 0.06	1.03 ± 0.04	0.85 ± 0.04	1.01	4.47
			OMC	27.68 ± 0.4	30.72 ± 0.3	25.48 ± 0.6	27.96	1.58
	Gervenne	Malaysia	BHA	1.93 ± 0.08	1.81 ± 0.06	1.72 ± 0.08	1.82	3.92
			BHT	n.d	n.d	n.d	n.d	n.d
			OMC	16.66 ± 0.4	14.61 ± 0.5	17.43 ± 0.4	16.23	2.68

Milk lotion	Nivea	Thailand	BHA	4.51 ± 0.12	4.46 ± 0.05	4.55 ± 0.04	4.50	1.57
			BHT	1.96 ± 0.09	2.58 ± 0.07	2.37 ± 0.06	2.30	3.21
			OMC	13.4 ± 0.26	12.5 ± 0.17	15.6 ± 0.21	13.83	1.55
	New Trendy	Malaysia	BHA	3.92 ± 0.15	4.15 ± 0.11	4.42 ± 0.09	4.16	2.82
			BHT	n.d	n.d	n.d	n.d	n.d
			OMC	7.82 ± 0.38	8.68 ± 0.32	10.48 ± 0.31	8.99	3.79
	Garnier	Indonesia	BHA	2.96 ± 0.09	2.47 ± 0.10	2.79 ± 0.09	2.74	3.32
			BHT	0.64 ± 0.03	0.83 ± 0.02	0.71 ± 0.03	0.73	3.26
			OMC	20.41 ± 0.38	16.64 ± 0.30	15.13 ± 0.30	17.0	1.86

Hair gel	De Boy	Malaysia	BHA	1.23 ± 0.05	1.27 ± 0.04	1.33 ± 0.04	1.28	3.14
			BHT	0.17 ± 0.01	0.24 ± 0.01	0.26 ± 0.01	0.22	3.40
			OMC	0.11 ± 0.01	0.15 ± 0.01	0.12 ± 0.01	0.13	4.52
	Beyond	Malaysia	BHA	1.28 ± 0.04	1.36 ± 0.06	1.49 ± 0.05	1.38	3.37
			BHT	0.13 ± 0.01	0.19 ± 0.01	0.16 ± 0.01	0.16	4.05
			OMC	0.31 ± 0.01	0.24 ± 0.01	0.36 ± 0.02	0.30	3.48
	Elite	Malaysia	BHA	1.42 ± 0.06	1.48 ± 0.03	1.63 ± 0.04	1.51	2.76
			BHT	0.17 ± 0.01	0.11 ± 0.01	0.23 ± 0.01	0.17	4.48
			OMC	0.81 ± 0.03	0.93 ± 0.02	0.79 ± 0.02	0.84	2.69

Hair oil	Elite	Malaysia	BHA	3.96 ± 0.04	3.93 ± 0.03	3.85 ± 0.05	3.89	1.06
			BHT	0.89 ± 0.02	0.87 ± 0.02	0.84 ± 0.01	0.87	2.11
			OMC	0.83 ± 0.02	0.82 ± 0.01	0.80 ± 0.01	0.82	1.37
	Gervenne	Malaysia	BHA	0.11 ± 0.01	0.12 ± 0.01	0.15 ± 0.01	0.13	4.66
			BHT	1.44 ± 0.05	1.61 ± 0.05	1.57 ± 0.06	1.54	3.25
			OMC	3.42 ± 0.06	3.29 ± 0.07	3.48 ± 0.05	3.40	1.75
	Johnsons	Philippines	BHA	0.34 ± 0.01	0.29 ± 0.01	0.26 ± 0.01	0.30	3.40
			BHT	0.19 ± 0.01	0.22 ± 0.01	0.14 ± 0.01	0.18	4.13
			OMC	0.51 ± 0.02	0.63 ± 0.01	0.56 ± 0.01	0.57	2.19

n.d: not detected or below detection limit.
